# The Survival of Psychobiotics in Fermented Food and the Gastrointestinal Tract: A Review

**DOI:** 10.3390/microorganisms11040996

**Published:** 2023-04-11

**Authors:** Patrycja Cichońska, Ewa Kowalska, Małgorzata Ziarno

**Affiliations:** Department of Food Technology and Assessment, Institute of Food Science, Warsaw University of Life Sciences-SGGW (WULS-SGGW), Nowoursynowska 159c St., 02-776 Warsaw, Poland; ewa_kowalska@sggw.edu.pl (E.K.); malgorzata_ziarno@sggw.edu.pl (M.Z.)

**Keywords:** psychobiotics, probiotics, bacterial viability, fermentation, gastrointestinal passage, brain health, neurological disorders, gut–brain axis

## Abstract

In recent years, scientists have been particularly interested in the gut–brain axis, as well as the impact of probiotics on the nervous system. This has led to the creation of the concept of psychobiotics. The present review describes the mechanisms of action of psychobiotics, their use in food products, and their viability and survival during gastrointestinal passage. Fermented foods have a high potential of delivering probiotic strains, including psychobiotic ones. However, it is important that the micro-organisms remain viable in concentrations ranging from about 10^6^ to 10^9^ CFU/mL during processing, storage, and digestion. Reports indicate that a wide variety of dairy and plant-based products can be effective carriers for psychobiotics. Nonetheless, bacterial viability is closely related to the type of food matrix and the micro-organism strain. Studies conducted in laboratory conditions have shown promising results in terms of the therapeutic properties and viability of probiotics. Because human research in this field is still limited, it is necessary to broaden our understanding of the survival of probiotic strains in the human digestive tract, their resistance to gastric and pancreatic enzymes, and their ability to colonize the microbiota.

## 1. Introduction

In the modern era, maintaining physical as well as emotional well-being is a constant struggle that many people tend to overlook. We are currently confronted with a plethora of information on mental illness that results in tragedies and rising suicide rates. Mental health and physical health are closely inter-related. The most important aspect that has piqued scientists’ interest in recent years is the gut–brain axis and, more specifically, the microbiota–gut–brain axis, because microbiota is the key element here [1]. The role of probiotic strains in alleviating mood disorders was first highlighted by an article published in the British Journal of Psychiatry in 1910. However, it was not until 1970 that scientific evidence became available when Bahr and his colleagues, based on their observation of piglets lacking microbiota, showed that the intestinal microbiota is in close communication with the brain, a distant topographic organ [2].

Probiotics allow for the modification of the function of intestinal microbiota. According to the latest FAO/WHO definition, probiotics are live microorganisms that, when administered in the right amount, have a beneficial effect on the health of the host [3]. The positive effect of probiotics on various types of digestive disorders was proven a long time ago, while recent data indicate that they also have an influence on the nervous system, immune system, hypothalamus, adrenal glands, or pituitary gland. Dinan et al. [4] proposed a definition of a psychobiotic that is merely a modification of the definition of a probiotic. According to this definition, psychobiotics are living organisms that, when administered in the right amount, have a beneficial effect in patients with mental or neurological diseases.

The central nervous system (CNS) is connected to the digestive system through the vagus nerve, the dorsal root ganglia of the spinal cord, and the autonomic nervous system of the intestines. The microbiota–gut–brain axis can act bidirectionally through hormones, cytokines, and bacterial metabolites secreted in the intestinal lumen. It has also been shown that the immunocompetence and neuroactivity of microbiota metabolites affect the structure and function of certain areas of the brain, such as the limbic system, which is responsible for emotions [5]. Studies that have been conducted so far clearly confirm the role of the vagus nerve, as well as the entire enteral nervous system, in the process of mood regulation. They have also revealed the relationship between intestinal dysbiosis and mental health deterioration. Dysbiosis, which refers to the imbalance between beneficial and pathogenic bacterial strains, affects the conversion of tryptophan to 5-hydroxytryptamine (serotonin), a neurotransmitter responsible for mood modulation and mental state. Insufficient serotonin synthesis may be one of the causes of mental disorders [6].

This paper describes the mechanisms of action of psychobiotics, their use in food products, and their viability and survival during gastrointestinal passage.

## 2. Characteristics of Psychobiotics and Their Impact on Human Health

**Figure 1:**
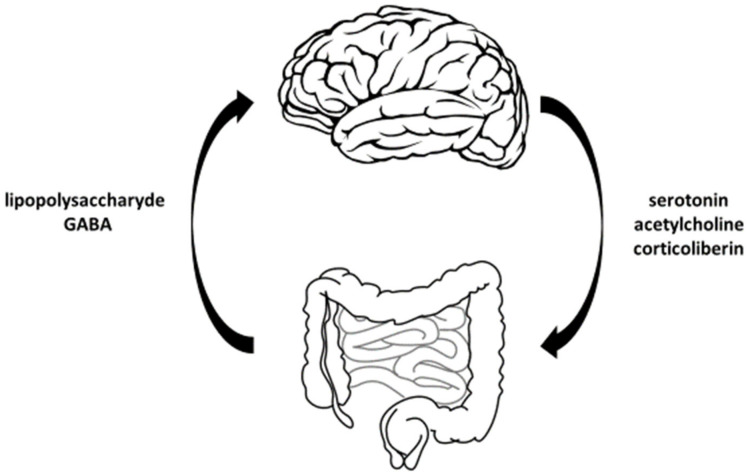
Graphical presentation of the bidirectionality of the gut–brain axis.

Intestinal micro-organisms indirectly affect the processes controlled by the CNS. This is achieved by modulating the immune system, the hypothalamic–pituitary–adrenal axis, neurotransmitters, and the synthesis of metabolites such as short-chain fatty acids (SCFAs), i.e., butyrate, acetate, and propionate. Data indicate that SCFAs greatly influence the microbiota–gut–brain axis and the physiology of the brain itself, due to their ability to penetrate the blood–brain barrier. They are also critical for the proper functioning of the intestinal barrier and maintaining its integrity [7]. The graphical presentation of the bidirectionality of the gut–brain axis is shown in Figure 1.

The current circumstances are not unfavorable and do not support maintaining good health. Some of the factors worsening the health of society are processed foods with large amounts of sugar, air pollution, the availability of stimulants, and a sedentary lifestyle. This article, however, focuses on another important factor—rapid deterioration of intestinal health and functioning. Intestines are home to superorganisms, i.e., the intestinal microbiota, which is a collection of micro-organisms, including bacteria, archaea, viruses, and eukaryotic organisms. The composition and functions of the intestinal microbiota affect the whole body as well as the work of each system [8]. For instance, these organisms play a key role in the process of digestion and deriving energy from food, as well as fermenting undigested food residues. The main source of energy for the intestinal microbiota is plant fiber, thanks to which the bacteria can produce metabolites, as well as mucus necessary for the intestinal epithelium [9,10]. The intestinal microbiota is also responsible for the synthesis of vitamins, including those from group B and vitamin K [11]. Therefore, when the homeostasis of the intestinal microbiota is disturbed, a state of intestinal dysbiosis is established. This has been shown to be associated with inflammatory bowel diseases (IBDs), irritable bowel syndrome (IBS), and the over-growth of bacteria in the small intestine, as well as civilization diseases, including obesity, diabetes, and cancer [12,13]. Furthermore, research in the last decade clearly indicates a link between mental illnesses such as depression and the intestinal microbiota [14].

Psychobiotics not only modulate the neuroimmune axes but also affect cognitive processes such as memory, learning, and general behavior. This discovery has led to changes in the current paradigm of symbiosis between bacteria and the human body. According to recent reports, this relationship resembles commensalism rather than pure symbiosis [15].

A crucial aspect that should be mentioned when describing the mechanisms of action of psychobiotics is the structure of the nervous system. Microglia are a collection of non-neuronal cells belonging to the CNS, which constitute 5–20% of glial cells. A particularly important fact in the context of the influence of the intestinal microbiota on mental health is that microglial cells release cytokines and, to some extent, are responsible for activating the inflammatory response [16]. The intestinal microbiota plays a role here, as it affects the maturation and function of the microglia. Studies on “germ-free” animals have revealed a longer microglial development process. This proves the existence of a mutual relationship between the microbiota and microglia, depending on the stage of development and the time of colonization by micro-organisms. Unfortunately, it is still unclear which specific mechanisms mediate the influence of the intestinal microbiota on microglia [17]. It is presumed that the specific modulation of microglia may be achieved only with the help of certain intestinal bacterial strains. These are mostly strains that have been recognized so far as psychobiotic. The psychobiotic effects of some probiotic strains have been demonstrated in both animal and human studies. Table 1 summarizes the most recent scientific reports on the possible uses of psychobiotics.

Studies indicate that strains producing high amounts of SCFAs have the greatest effect on microglia and can also restore the changes caused in the microglia of “germ-free” mice [59]. The psychophysiological effects of psychobiotics include systemic effects on the hypothalamic–pituitary–adrenal axis and the glucocorticoid response, as well as on the inflammation itself, which is caused by inflammatory cytokines and, more specifically, their abnormal concentration [60]. Pro-inflammatory cytokines, such as interferon α, can cause mental illnesses, such as depression. This has been confirmed by studies on rats and mice, based on numerous experiments [61,62,63]. However, human studies are limited for safety reasons. In a study involving 124 participants (both men and women), one half of the study group consumed a fermented milk beverage containing the probiotic strain *Lactobacillus casei* Shirota for 3 weeks, while the other half received a placebo. People in the study group who described themselves as depressed at the beginning of the study rated themselves as much happier at the end of the experiment. This could point to the beneficial effects of psychobiotic consumption [44].

As mentioned earlier, one of the factors disrupting the functioning of the intestinal microbiota is stress, which is also directly related to an increased frequency of mental problems. In a study by Ma et al. [21], a 12-week supply of psychobiotics increased the diversity of intestinal microbiota. The authors indicated that the bacteria whose number increased were responsible for the production of SCFAs and gamma-aminobutyric acid (GABA). Psychobiotics, by modulating the function and composition of the intestinal microbiota, can alleviate nervous tension and anxiety in adults, through the microbiota–gut–brain axis [21].

Chronic stress is also associated with elevated levels of cortisol, which is commonly known as the stress hormone. Cortisol is a steroid hormone produced by the adrenal cortex and belongs to the group known as glucocorticosteroids—compounds that are involved in the regulation of glucose levels in the human body. All nervous, hormonal, and circulatory messages reach the intestines through the gut–brain axis. These messages induce the activation of mast cells and changes in the functions of the intestinal barrier. Activation of the autonomic nervous system causes an increase in the concentration of cortisol and pro-inflammatory cytokines, such as tumor necrosis factor-alpha, interleukin-8, or interleukin-6. In such cases, psychobiotic intervention can be very helpful in reducing the blood cortisol concentration [64].

Certain groups of workers, including those employed in the information technology (IT) industry, are particularly affected by stress and work-related emotional tension. Often, this population is at a greater risk of developing diabetes, high blood pressure, and depression. Specific psychobiotic strains can relieve stress and improve overall mood. For instance, the effectiveness of *Lactiplantibacillus plantarum* PS128TM strain was tested in an 8-week intervention in IT workers. This short-term psychobiotic therapy improved participants’ overall self-esteem, decreased blood cortisol levels, and reduced sleep disturbances and night-time awakenings [24].

## 3. Psychobiotics in Fermented Food

In recent times, consumers have shown great attention toward foods that have additional health benefits [65,66]. This has resulted in the growing popularity of functional food, which is often defined as food that, in addition to its nutritional effect, significantly affects the functions of the body, improving health and well-being and/or reducing the risk of diseases [67]. Food is no longer intended to only satisfy hunger and provide necessary nutrients for humans but also offer health benefits that may reduce disease risks, as well as promote optimal wellness and improve both physical and mental well-being [68,69]. This can be attributed to consumers’ awareness of the deterioration of health caused by a busy lifestyle, poor food choices, and low physical activity [70]. Among functional foods, fermented products, which are carriers of probiotic strains with a multidirectional impact on human health, are particularly popular [71].

### 3.1. Food Fermentation

Fermentation is a process used by humans since the need for extended food storage arose. Various food preservation techniques, including fermentation, were followed in the Neolithic period approximately 10,000 years ago [72]. Fermentation is an anaerobic process that converts sugars to other ingredients while generating energy for the micro-organism or cell [73]. This process helps to preserve food by lowering its pH and producing antimicrobial ingredients such as organic acids (lactic acid, acetic acid, formic acid, and propionic acid), ethanol, carbon dioxide, diacetyl, reuterin, and bacteriocins [74,75]. In addition, the taste and texture of the product are changed from those of the starting materials [74,76].

Fermentation is used worldwide to produce a wide range of products, either spontaneously from original ingredients and environment or in a controlled manner through the addition of specific starter cultures [77,78]. The type of fermented food produced most often depends on community food habits and the availability of plant or animal sources [75,79]. Therefore, various species of microorganisms are used in fermentation and produce substances that shape the properties of the final products. In particular, bacteria, yeasts, and molds are used, which, due to their enzyme portfolio, can carry out different types of fermentation (e.g., lactic, alcohol, butter, propionic) [80,81].

Among bacteria, lactic acid bacteria (LAB) and bifidobacteria are currently used worldwide to produce various types of fermented products, including fermented milk (such as yogurt, kefir, cheese, ice cream) [82,83], sauerkraut [84], kimchi [85], fermented meat and fish [86,87], fermented cereals [88,89], and traditional dishes [73,90]. LAB includes bacteria such as *Lactobacillus*, *Streptococcus*, *Enterococcus*, *Lactococcus*, *Pediococcus*, and *Leuconostoc*. Bifidobacteria are often described with LAB because they are also anaerobic Gram-positive bacteria and produce lactic acid as a major product of carbohydrate metabolism [91,92]. Fermentation with LAB and bifidobacteria converts pyruvate molecules from glycolysis into lactate [73].

Alcoholic fermentation is mainly carried out with yeasts, which are widely applied in baking and brewing [93]. This process converts sugars into ethanol and carbon dioxide [75]. Yeasts of the genus *Saccharomyces* are often used, e.g., for leavening dough during the production of bread, as well as in the production of beer [94], wine [95], and some fermented milk products (e.g., kefir) [96]. Molds are frequently used in fermentation mainly due to their ability to produce enzymes (such as α-amylase, acid/alkaline proteases, amyloglucosidase-galactosidase, cellulase maltase, invertase, lipases) and reduce antinutrients [75]. The micro-organisms involved in fermentation also exhibit probiotic properties and are classified as “Generally Recognized As Safe (GRAS)” by the US Food and Drug Administration [97,98].

Food fermentation extends shelf life, promotes food safety, increases the nutritional value, and shapes the taste and texture of products, due to the activity of the microbial ecosystem [78,99]. Fermented foods have gained popularity among consumers for their beneficial effects on human health. This is mainly related to the modification of the intestinal microflora, as well as the positive health effect of fermentative metabolites, such as lactic acid, vitamins, and exopolysaccharides [78,100]. It has been reported that fermentation increases the antioxidant, anti-inflammatory, antiapoptotic, and anticancer properties of food [75,101,102]. Moreover, microbial enzymes involved in fermentation reduce the content of antinutrients (such as phytates, tannins, oligosaccharides) in food, which, in turn, increases the bioavailability of protein and vitamins [103].

**Figure 2:**
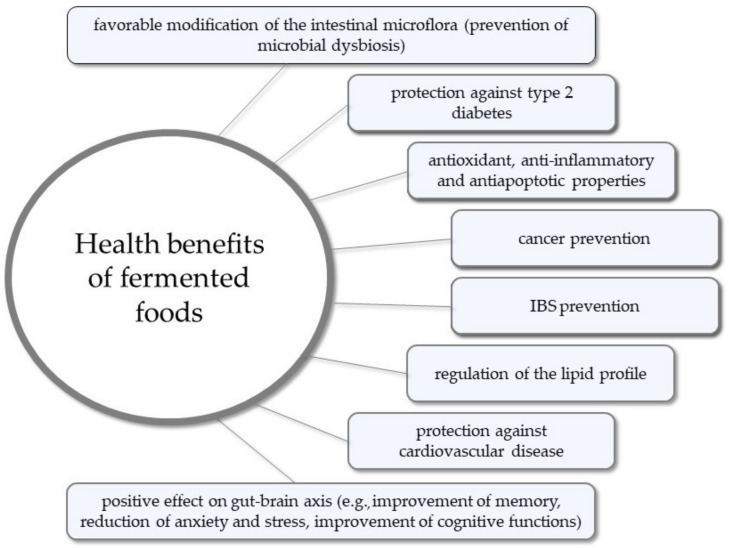
Health benefits of consuming fermented foods.

Consumption of fermented foods can also prevent microbial dysbiosis, regulate the lipid profile, and protect against IBS, IBDs, cardiovascular disease, type 2 diabetes, and some types of cancers such as bladder and colorectal cancer [74,98,104]. There is also growing evidence that fermented foods have an influence on the gut–brain axis and, consequently, neurological and mental health conditions [105,106]. The health benefits of consuming fermented foods are summarized in Figure 2.

### 3.2. Effect of Fermented Foods on the Gut–Brain Axis and Brain Health

Consumption of fermented foods can result in some modifications of the gut microflora, which can have a direct impact on brain health. This is mediated by various mechanisms, including the effect of microbiota on the production of neurotransmitters (e.g., GABA, noradrenaline, serotonin, dopamine, acetylcholine), direct activation of neural pathways between the gut and brain (vagus and enteric nervous system), modulation of neurotrophic chemicals (e.g., brain-derived neurotrophic factor—BDNF) and immune cells (e.g., interleukin-1, interferon), and analgesic properties [107,108].

Intestinal microbes produce essential molecules with neuroactive functions that affect the gut–brain axis [107]. One of these molecules are SCFAs, such as butyrate, acetate, and propionate, which are produced during microbial fermentation mainly from dietary fiber in the gut. SCFAs are known to have neuroactive properties and an essential function in the CNS [75,107,109]. Moreover, the products of food fermentation enhance the neuroprotective effects of SCFAs by increasing their bioavailability through intestinal absorption and the utilization of ingested nutrients within the body. Thus, the gut microbiota also regulates the absorption of phytochemicals and their anti-inflammatory and antioxidant functions [75,110].

Sustained colonization of the gut microbiome by beneficial probiotic micro-organisms contributes to the gut–brain relationship [111,112]. Communication between the gut and brain is stabilized through the nervous system, including the vagus nerve, and this in turn regulates the physiological functions of metabolism, digestion, assimilation, indulgence, immunity, and stress reactions [98]. The gut–brain axis moderates the coordination between the brain, the intestinal tract, and the endocrine and immune systems, which play a role in maintaining gut functions. Disruptions in this axis have been associated with psychiatric symptoms, such as anxiety; functional gastrointestinal disorders, such as IBS; and neuroimmunological disorders [106,109]. The consumption of fermented foods can, therefore, affect the complex relationship between the gut microbiota and the brain. Several animal and human studies have proven the influence of fermented foods on neurological disorders, behavior, and mood. 

#### 3.2.1. Animal Studies

Some animal studies have examined the effects of consumption of both plant- and animal-based fermented foods on neurological disorders. Musa et al. [113] analyzed the neuroprotective (anti-inflammation) effect of cow milk fermented with *Limosilactobacillus fermentum* LAB9 or *L. casei* LA-BPC in mice with induced neuronal inflammation. Mice fed with fermented milk for 28 days showed a significant improvement in nitrosative stress, attenuation of memory deficit caused by neuronal inflammation, and an increase in antioxidants, as well as a reduction in lipid peroxidation and the levels of acetylcholinesterase and pro-inflammatory cytokines. The results indicated that the consumption of fermented milk may contribute to alleviating nerve inflammation and memory deficit caused by induced neuronal inflammation [113]. 

In a study by Van de Wouw et al. [114], two distinct kefirs (Fr1 and UK4, with microflora dominated by *Lactococcus lactis*), or unfermented milk control, were administered to mice. Consumption of both types of kefir caused a significant change in the composition and functional capacity of the host microbiota and enhanced the GABA-producing ability of the gut microbiota. The intake of Fr1 ameliorated the stress-induced decrease in serotonergic signaling in the colon, while UK4 ameliorated stress-induced deficits in reward-seeking behavior and increased fear-dependent contextual memory [114].

Woo et al. [115] investigated the efficacy of kimchi (KME) and its bioactive compounds in ameliorating amyloid beta(Aβ)-induced memory and cognitive impairments. Mice treated with KME bioactive compounds and KME methanolic extract for 2 weeks showed a restoration of Aβ-induced cognitive deficits. The groups treated with KME and bioactive compound groups showed increased expression of antioxidant enzymes but decreased expression of inflammation-related enzymes. The authors concluded that due to its antioxidative and anti-inflammatory properties, kimchi rich in bioactive compounds might help to attenuate the symptoms of Alzheimer’s disease (AD) [115].

Wu et al. [116] studied the effect of adzuki bean sprout milk fermented with *Streptococcus thermophilus*, *Lactobacillus bulgaricus*, *L. plantarum*, and *Levilactobacillus brevis* J1 in a chronic depression mouse model. Mice treated with fermented product showed an increase in the levels of 5-hydroxytryptamine, norepinephrine, and dopamine in the hippocampus. The authors indicated that treatment with fermented plant milk can reduce and possibly prevent mild depression-like symptoms in mice by increasing social interaction and enhancing the pleasure derived from movement [116].

Several studies also describe the effects of fermented soy and its products on brain health. For instance, Go et al. [117] investigated the effects of soybean products (Cheonggukjang) fermented with *Bacillus subtilis* MC31 and *Latilactobacillus sakei* 383 on induced cognitive defects in mice. After 4 weeks of administration of Cheonggukjang at various doses, the mice showed a significant improvement in induced short-term and long-term memory loss. In addition, a decrease in the number of dead cells was observed in the granule cell layer of the dentate gyrus, as well as a dose-dependent increase in nerve growth factor concentration and an increase in superoxide dismutase activity, which may be beneficial in the treatment of neurodegenerative diseases, including AD, Parkinson’s disease, and Huntington’s disease [117].

Yoo and Kim [118] studied the effectiveness of defatted soybean powder fermented with *Lactiplantibacilluspentosus* var. *plantarum* C29 in the protection against scopolamine-induced memory impairment in mice. Fermented soybean powder caused an increase in BDNF expression in the hippocampi of scopolamine-treated mice and inhibited acetylcholinesterase activity in vitro and ex vivo, which indicates that the fermentation process might increase the ameliorating effect of soybean against memory impairments [118]. Similarly, Lee et al. [119] determined whether *L. plantarum* C29-fermented defatted soybean could attenuate memory impairment in transgenic mice. The authors observed increased cognitive function and suppressed Aβ expression in mice fed with fermented soy [119].

#### 3.2.2. Human Studies

Human studies have shown that the consumption of fermented foods in general as well as foods fermented with individual bacterial strains can lead to an improvement in neurological disorders and behavior. Kim and Shin [120] conducted a cross-sectional analysis of a large, nationwide, population-based database to evaluate the association between probiotic food consumption and depression status. The population included in the analysis comprised 26,118 individuals aged 19–64 years. The authors observed that compared with the lowest tertile of probiotic food consumption, the highest tertile had significantly lower odds of depression severity and self-reported clinical depression. Although no significant association between probiotic food consumption and clinical depression was observed in women, men showed a significantly lower prevalence of clinical depression in the highest tertile [120]. In a randomized, double-blind controlled study by Ohsawa et al. [121], healthy, middle-aged adults who consumed *Lactobacillus helveticus*-fermented milk drink for 8 weeks (190 g/day) showed a significant improvement in attention score and delayed memory score compared to the placebo group [121]. Mohammadi et al. [122] studied the effects of probiotic yogurt (containing *L. acidophilus* LA5 and *Bifidobacterium lactis* BB12) and multispecies probiotic capsule (containing *L. casei*, *L. acidophilus*, *Lacticaseibacillus rhamnosus*, *L. bulgaricus*, *Bifidobacterium breve*, *Bifidobacterium longum*, *S. thermophilus*) supplementation (100 g/day, for 6 weeks) on mental health in petrochemical workers (n = 70). After the intervention, a significant improvement in general health, as well as in depression, anxiety, and stress scale scores, was observed in the probiotic yogurt group and probiotic capsule group, while there was no significant improvement in the conventional yogurt group [122]. A study by Tillisch et al. [123] examined the effect of the consumption of milk fermented with *Bifidobacterium animalis* subsp. *lactis*, *S. thermophilus*, *L. bulgaricus*, and *L. lactis* subsp. *lactis* on intrinsic connectivity in the brain or responses to emotional attention tasks. The consumption of probiotic-fermented milk for 4 weeks in healthy women resulted in a reduced task-related response of a distributed functional network involving affective, viscerosensory, and somatosensory cortices, i.e., affected activity of brain regions that control central processing of emotion and sensation [123].

The consumption of fermented food may influence regulation of stress hormones and, thus, reduce the perception of stress in humans [75]. Berding et al. [124] investigated the effect of a psychobiotic diet (rich in prebiotic and fermented foods) on the microbial profile and function as well as mental health outcomes in a healthy human population. Forty-five adults were randomized and received a psychobiotic (n = 24) or control (n = 21) diet for 4 weeks. The psychobiotic diet resulted in a reduction in perceived stress, particularly in those who were most adherent to the diet, and greater changes in perceived stress scores associated with volatility in microbial function [124]. Nishihira et al. [125] conducted a placebo-controlled, randomized, double-blind clinical trial with yogurt containing *Lactobacillus gasseri* SBT2055 and *B. longum* SBT2928 to test its immunomodulatory and stress-related effects. The study involved healthy adult volunteers who consumed 100 g/day of probiotic yogurt (n = 115) or placebo yogurt (n = 109) for 12 weeks. The group consuming fermented yogurt showed a higher number of natural killer cells, as well as a significant decrease in adrenocorticotrophic hormone, which indicates improved immunity and alleviation of stress [125].

Studies with humans also indicate the effect of fermented food consumption on academic stress. Kato-Kataoka et al. [126] conducted a pilot study investigating the effects of the probiotic *L. casei* Shirota (LcS) on stress responses in medical students. Two groups (24 tested and 23 placebo participants) consumed either a fermented milk with LcS (100 mL once a day) or a placebo milk for 8 weeks. One day before the examination, the placebo group showed a significant increase in salivary cortisol and plasma L-tryptophan, along with a significant increase in anxiety. The number of subjects experiencing common abdominal and cold symptoms and the total number of days each subject experienced these physical symptoms were significantly lower in the LcS group than in the placebo group [126]. Academic stress was also evaluated in a group of medical students, before and after ingestion of an aguamiel-based beverage fermented with *Lacticaseibacillus paracasei*, *L. plantarum*, and *L. brevis* (n = 27), and a control group (n = 18). The results showed that the consumption of 100 mL of a fermented beverage for 8 weeks led to a significant reduction in academic stress, while there were no significant changes in the perception of academic stress in the control group (placebo intervention). Moreover, consumption of the fermented beverage significantly increased the phyla *Firmicutes* and *Bacteroidetes* but not *Gammaproteobacteria* in the microbiota of the subjects [127].

Probiotics and prebiotics (defined as substrates that are selectively utilized by host micro-organisms, eliciting health-beneficial effects) can cause an improvement in cognitive functions in patients with mild cognitive impairment (MCI) [92,128]. There are also reports indicating a similar effect of fermented foods. Handajani et al. [129] studied the effects of 6-month tempeh consumption on global cognition in MCI patients who were 60 years of age or older. The study involved a total of 90 subjects, who were divided into three groups: group A (consumed 100 g of Tempeh A/day), group B (consumed 100 g of Tempeh B/day), and group C (control). An increase in global cognitive scores was found in groups A and B, suggesting that both Tempeh A and Tempeh B were effective in improving global cognitive function in older people with MCI [129]. In a study by Hwang et al. [18], a population of 100 individuals with MCI consumed *L. plantarum* C29-fermented soybean (800 mg/day, n = 50) or placebo (800 mg/day, n = 50) for 12 weeks. Compared to the placebo group, the group that consumed fermented soybean showed a greater improvement in combined cognitive functions, and this effect was associated with increased serum BDNF levels [18].

Studies suggest that the consumption of fermented foods can contribute to an improvement in cognitive deficits associated with neurological diseases, including AD. Ton et al. [130] investigated the effects of supplementation (2 mL/kg/day) with probiotic fermented milk with kefir grains for 90 days in AD patients with cognitive deficit. The subjects showed a marked improvement in memory, visual–spatial/abstract abilities, and executive/language functions. At the end of the intervention, there was an absolute/relative decrease in the levels of several cytokine inflammation markers and oxidative stress markers, as well as an improvement in serum protein oxidation, mitochondrial dysfunction, DNA damage/repair, and apoptosis [130]. Akbari et al. [131] assessed the effect of supplementation with 200 mL/day probiotic milk containing *L. acidophilus*, *L. casei*, *Bifidobacterium bifidum*, and *L. fermentum* on cognitive function and metabolic status in 60 AD patients. After 12 weeks of intervention, patients treated with probiotic milk showed a significant improvement in the mini-mental state examination score, compared with the control group. This indicates that the consumption of probiotic milk led to an improvement in cognitive function [131].

All the above-described studies indicate that fermented foods exert a positive effect on various aspects of brain health, by, for example, improving memory, reducing anxiety and stress, enhancing cognitive functions, and affecting the immune, hormonal, and antioxidant parameters of the body. Therefore, regular consumption of fermented food can help to prevent neurological disorders and may also be considered a nutritional therapy supporting the pharmacological treatment of neurodegenerative diseases and depression.

## 4. Survival of Psychobiotics in Fermented Food

The ingestion of fermented foods results in a potential increase in the numbers of microbes in the diet by up to 10,000-fold. A diet based on fermented foods is contradictory to the typical Western diet, which is rich in highly processed and sanitized foods [74,132]. Fermented foods have a high potential to carry probiotic strains, including psychobiotic ones [98,133]. However, it is important that the micro-organisms in these foods remain viable at a concentration of about 10^6^–10^9^ CFU/g or mL during processing, storage, and even digestion [74,83]. The use of probiotics in food products is associated with technological and therapeutic challenges since probiotic viability is an important factor affecting the biological effects of these products on the consumers. Products containing probiotics must meet stringent criteria to ensure that probiotic survival is maintained from large-scale industrial production until consumption [83,134].

The survival of bacteria in a product is influenced by many factors, including those related to the type of food (e.g., pH, macronutrient content, water activity, presence of natural antibiotics) as well as the conditions of production and storage (e.g., time, temperature, inoculation rate, oxygen content, packing materials) [83,135,136,137]. Therefore, when designing psychobiotic products, it is extremely important to monitor bacterial viability and maintain it at an appropriate level so that the products offer therapeutic benefits to humans [133]. The matrix of fermented products is critical for the survival of bacteria, which may either favor or interfere with their long-term activity [74]. Dairy products are the most popular carriers of psychobiotics in food, but their survival has also been analyzed in plant matrices in recent times [108,138,139]. Table 2 shows the survival of psychobiotics in different types of fermented foods.

### 4.1. Survival of Psychobiotics in Dairy Products

Dairy products are considered to be excellent carriers for delivering probiotic bacteria to the human gastrointestinal tract [167]. Yogurt, fermented milk drinks, cheese, or ice cream are some of the dairy products that are often used for carrying probiotics [168,169]. This is mainly due to the physicochemical composition of these products, which includes a large amount of proteins, fats, and lactose, as well as their high buffering capacity. These factors enable the protection of probiotic bacteria during their passage through the gastrointestinal tract [75,83].

As shown in Table 2, dairy products also provide a good matrix for the development of probiotic psychobiotics. A study showed that after 20 h of fermentation of cow milk with *L. rhamnosus* GG, a viable cell count of 2.4 × 10^9^ CFU/mL was achieved. Milk supplementation with ingredients that are essential for *L. rhamnosus* GG (cysteine, serine, arginine, proline, aspartic acid, glutamic acid, guanine, uracil, and xanthine) made it possible to increase the viable cell count to 4.6 × 10^9^ CFU/mL [142]. A similarly high and viable population of *L. rhamnosus* GG was achieved after 48 h of fermentation of cow milk (8.0 log CFU/mL) [143] and 18 h of fermentation of goat milk encapsulated in buttermilk proteins (9.3 log CFU/mL) [144]. For the fermentation of goat milk, buttermilk protein was used as a thermoprotector for the probiotic cells undergoing the spray-drying process.

In fermented dairy products, psychobiotics reach a therapeutic dose of micro-organisms (above 10^6^ CFU/mL) not only directly after the fermentation but also after storage. Fermentation of yogurt and cheese with *L. plantarum* 299v resulted in a cell count of 8.0 and 9.5 log CFU/mL, respectively. After 56 days of storage, the bacterial count of psychobiotic cells in yogurt increased to 7.5 log CFU/mL, while in cheese, it was 9.0 log CFU/mL [140]. Probiotic ice cream fermented with *L. plantarum* ATCC 8014 had a cell count of 7.55 log CFU/mL after fermentation and 7.65 log CFU/mL after 60 days of storage. In addition, the viable population of bacteria in these products was increased by the addition of coconut residue fiber (0.03 g/mL), which resulted in a cell count of 7.75 log CFU/mL after fermentation and 8.1 log CFU/mL after storage [141]. In milk ice cream fermented with *L. rhamnosus* GG, the cell count was 8.3 log CFU/mL after fermentation and 7.3 log CFU/mL after 90 days of storage. In this case, the addition of dietary fiber did not significantly increase the population of micro-organisms in the product [145].

High survival of psychobiotics has also been reported for various types of fermented milk. Zamberlin and Samaržija [146] fermented sheep milk with *L. rhamnosus* GG by applying different milk thermal treatments. In the probiotic yogurt that was produced by using the nonstandard heat treatment of milk (60 °C/5 min), the bacterial cell count after fermentation was 7.5 log CFU/mL, and after 21 days of storage, it was 7.4 log CFU/mL, while in the yogurt produced by using the standard heat treatment of milk (95 °C/5 min), the counts were 8.0 and 7.8 log CFU/mL, respectively [146]. Lavrentev et al. [151] fermented skimmed milk containing milk protein concentrate with *Bacillus coagulans* MTCC 5856. The cell count was 8.4 log CFU/mL after fermentation and 8.1 log CFU/mL after 60 days of storage. The authors concluded that fermented milk is an excellent carrier of *B. coagulans* [151]. Fermented milk is also a good carrier for the psychobiotic *L. casei* Shirota. Sumalapao et al. [148] fermented cow milk with *L. casei* Shirota and achieved a viable cell count of 3.64 × 10^8^ CFU/mL after fermentation and 2.65 × 10^8^ CFU/mL after 31 days of storage, while Angmo et al. [149] achieved 8.8 log CFU/mL after fermentation and 7.8 log CFU/mL after 28 days of storage.

Psychobiotic micro-organisms can also be found in dairy desserts. In a milk-based dessert (2.7% fat) with cranberry sauce fermented with *L. casei* Shirota, a cell count of 8.0 log CFU/mL was observed after fermentation and 7.3 log CFU/mL after 21 days of storage [147]. A milk pudding fermented with *L. casei* Shirota had a cell count of 7.3 log CFU/mL after fermentation and 9.0 log CFU/mL after 28 days of storage [150]. These results indicate that a wide range of dairy products can serve as excellent carriers for psychobiotics, which show high survival not only after the fermentation process but also after storage.

### 4.2. Survival of Psychobiotics in Plant Products

The consumption of probiotic micro-organisms with dairy products has some disadvantages, including the presence of allergens (e.g., lactose, casein) and a high content of fat and cholesterol [75,167]. This has led to the introduction of new products based on nondairy matrices such as fruits, vegetables, legumes, and cereals. These alternative products can provide a good matrix for the development of probiotics (including psychobiotics) since they contain nutrients such as minerals, vitamins, dietary fibers, and antioxidants [75,167,170]. However, some nondairy matrices exhibit unfavorable properties such as high acidity (e.g., in fruit juices) or low water activity, which may affect probiotic development [83]. Furthermore, designing plant-based probiotic products is a great challenge, as these products should have the same survival rate of micro-organisms as in dairy products in order to exhibit therapeutic properties [171].

Probiotics in plant matrices should also be protected against acidic conditions. This can be achieved by microencapsulation technologies, in which cells are entrapped into matrices with a protective coating [167,172]. Giordano et al. [159] applied microencapsulation for *L. reuteri* DSM 17938 to inhibit changes in a probiotic tomato juice. The authors observed a cell count of 7.1 log CFU/mL after fermentation and 5.7 log CFU/mL after 28 days of storage in tomato juice, and these values increased to 7.6–7.8 log CFU/mL after storage with microencapsulation of micro-organisms [159]. Mirkovic et al. [158] prepared fermented dark chocolate with encapsulated *L. plantarum* 299v. After fermentation, the viable cell count was 8.2 log CFU/mL, and after 360 days of storage, it was 5.5 log CFU/mL. The count remained above 7 log CFU/mL until 90 days of storage. Based on these observations, the authors concluded that encapsulated *L. plantarum* 299v could be successfully used in the production of probiotic dark chocolate [158].

Even without encapsulation, a therapeutic viable population level of psychobiotics can be achieved in plant-based foods. A study showed that 24 h fermentation of blueberry pomace by using *L. rhamnosus* GG resulted in a viable cell count of 11.6 log CFU/mL [161]. Similarly, 24 h fermentation of sesame with *L. plantarum* P8 resulted in a viable cell count of 8.6 log CFU/mL [154]. It has been shown that *L. plantarum* P8 is an effective psychobiotic for the fermentation of whole soybeans, and after 24 h of fermentation, the bacterial survival rate was 10.5 log CFU/mL [155].

The literature data indicate that fruit juices are a good carrier for psychobiotics. Fermentation of pineapple and jussara juice with *L. rhamnosus* GG resulted in a viable cell count of 7.2 log CFU/mL, and after 28 days of storage at 8 °C, the count increased to 7.7 log CFU/mL [163]. Zoghi et al. [153] studied apple juice supplemented with different levels of fructo-oligosaccharide, ascorbic acid, and citric acid and subsequently fermented with *L. plantarum* ATCC 8014. Regardless of the concentration of additives, a cell count of 11.0–11.5 log CFU/mL was achieved after fermentation, and a cell count of 7.7–8.6 log CFU/mL was achieved after 6 weeks of storage [153]. Amanda and Choo [152] studied the fermentation of watermelon juice by using *L. plantarum* ATCC 8014 and observed that after fermentation, the viable cell count was 8.8 log CFU/mL and remained at about 11.0 log CFU/mL even at week 2 of refrigerated storage at 4 °C [152].

Currently, various types of nondairy fermented beverages are gaining popularity, which can be considered to be convenience foods rich in probiotics [173,174]. They can also be a carrier of highly viable psychobiotic micro-organisms. Fermentation of a teff-based beverage with *L. rhamnosus* GG resulted in a viable cell count of 8.1 log CFU/mL after fermentation and 7.8 log CFU/mL after 25 days of refrigerated storage [164]. Coconut milk fermented with *L. reuteri* DSM 17938 showed a cell count of 8.6 log CFU/mL, which remained stable during 30 days of storage [160], while coconut water fermented with *B. coagulans* MTCC 5856 had a count of 9.73 log CFU/mL after fermentation [166]. Fermentation of hazelnut milk with *L. rhamnosus* GG resulted in a cell count of 7.9 log CFU/mL after fermentation and 8.3 log CFU/mL after 28 days of cold storage [162]. Coffee brews fermented with *L. rhamnosus* GG had a cell count of 7.8 log CFU/mL after fermentation and 7.0 log CFU/mL after 7 weeks of storage at 4 °C. After 7 weeks, the number of *L. rhamnosus* GG drastically decreased in the tested product. However, in the coffee brews fermented with a mixed culture of *L. rhamnosus* GG and *Saccharomyces boulardii* CNCM-I745, the cell count of *L. rhamnosus* GG was maintained at 7.0 log CFU/mL for 14 weeks of storage. This indicated that yeasts can effectively enhance probiotic bacterial viability in coffee brews and may, thus, be useful for formulating shelf-stable probiotic food products [165].

Bread is an interesting nondairy vehicle for probiotics given its daily consumption worldwide. However, probiotic incorporation in this product is challenging due to the application of high baking temperatures. Zhang et al. [156] analyzed the influence of various baking conditions and subsequent storage on the survival of *L. plantarum* P8, which exhibits psychobiotic properties. Under all baking conditions, the probiotic viability decreased from 10^9^ to 10^4–5^ CFU/g after baking; however, after 5 days of storage, the cell viability in bread crust was 8.0 log CFU/mL [156]. Supasil et al. [157] studied the characteristics of sourdough and sourdough bread prepared by using fermented water from Asian pears and Assam tea leaves with *L. plantarum* 299v and *Saccharomyces cerevisiae* TISTR 5059 as starter cultures. The authors noted that fermented water with the studied cultures improved dough fermentation and bread quality, and the viable cell count of psychobiotic *L. plantarum* in sourdough was 7.9 log CFU/mL [157].

The development of novel, economic, and technological matrices is essential to design non-diary probiotic products for consumers who avoid dairy products [75]. The cited studies indicate that plant products can be a good matrix for probiotics exhibiting psychobiotic properties, but the viability of probiotic bacteria depends on their species and strain. Further research is needed to understand the survival of probiotics in various plant matrices.

## 5. Survival of Psychobiotics in Human Gastrointestinal Tract

**Figure 3:**
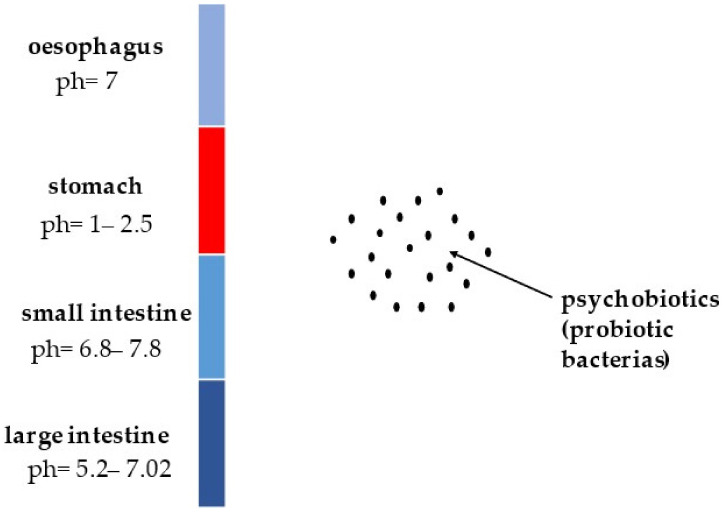
Graphical presentation of the conditions in the digestive system to which psychobiotics are exposed.

The key objective of studies on probiotics is to control their activity and survival during the gastrointestinal passage. Such studies are carried out mainly on laboratory animals, such as mice and rats, but often, model digestive systems that accurately reproduce the conditions in the human digestive tract are also used. The results from these studies are crucial for classifying a given strain under the group of probiotics and using it in targeted probiotic therapy. A graphical presentation of the variety of conditions in the digestive system to which psychobiotics are exposed is shown in Figure 3 [12].

Unfortunately, there is limited research on the gastrointestinal survival of these less common psychobiotic strains. The available studies should be supplemented with new ones to make the best use of psychobiotics. The findings of some studies on the survival of selected psychobiotic strains in model digestive systems and clinical trials on humans are presented below.

Strains of the species *B. animalis*, *S. thermophilus*, and *Lactobacillus delbrueckii* subsp. *bulgaricus* have been studied for their psychobiotic effects and their potential to produce increased amounts of GABA, a neurotransmitter involved in mood modulation. Although studies have shown a significantly increased diversity of the intestinal microbiota, the intake of these bacterial strains resulted in an increase in the population of *Bacteroidetes*, which are mainly responsible for the anti-inflammatory functions of the intestinal microbiota. All the tested strains were detectable after 48 h in the bioreactors, but a decrease in their population was observed. Statistical analysis, however, showed that this decrease was not statistically significant [175].

One of the better-studied probiotic strains, with a strong effect on the microbiota–gut–brain axis, is *L. plantarum* 299v. This strain has long been used in the probiotic treatment of many digestive diseases, such as IBS, as well as intestinal dysbiosis and abnormal iron absorption. The organism also remains viable in unfavorable conditions, such as in gastric juice supplemented with a proton pump inhibitor—pantoprazole. Despite the increased secretion of gastric juice, after a week of probiotic intervention, *L. plantarum* 299v bacteria were observed in stool samples, which implies their resistance to the acidic environment in the stomach [176]. This strain can survive even in extremely unfavorable conditions of the digestive tract and successfully adapt to the conditions in the large intestine, where it can persist for a long period of time. In a study by Goossens et al. [177], *L. plantarum* was isolated from human stool samples 8 days after the last administration of a probiotic fruit drink containing this strain [177].

Another most widely described probiotic strain is *L. rhamnosus* GG (LGG). In addition to the recently discovered psychobiotic effect, this strain is most often used in children and adults to support the intestinal microbiota during antibiotic therapy, as well as to treat traveler’s diarrhea, *Clostridium difficile* infections, IBS, and IBDs. Similar to the previously described strain, *L. rhamnosus* GG shows a very high survival rate during the gastrointestinal passage. To improve its activity, it is often encapsulated with various materials, such as pectin or sodium alginate. The initial number of unencapsulated *L. rhamnosus* GG was 9.76 log CFU/mL, and after 4 h in a simulated colonic system, the number of viable bacterial cells decreased to 3.25 log CFU/mL. However, even a slight addition of glucose or pectins to the bacterial suspension resulted in an increase in the number of active probiotics to 5.36–6.52 log CFU/mL [178].

Many scientific publications describe the effect of the *L. casei* Shirota strain. The first reports on this strain appeared in the PubMed database in 1998. The strain has been proven to be beneficial for upper respiratory tract infections. Interestingly, Shirota helps in treating digestive tract dysfunctions that occur in professional athletes due to intense training [179]. It exerts a wide spectrum of action on the microbiota–gut–brain axis as follows [179]:

- lowers the concentration of cortisol in the blood,
- relieves stress,
- reduces anxiety, and
- soothes the symptoms of irritable bowel syndrome.

One of the most popular studies on the *L. casei* Shirota strain is a clinical trial conducted on a population of healthy Chinese subjects who were given a probiotic drink containing 10^8^ CFU/mL for 2 weeks. The presence of the strain was confirmed via the culture method followed by ELISA. The strain showed excellent survival during gastrointestinal transit. The number of viable cells in the recovered fecal samples was 6.86–7.17 log CFU/mL, indicating the high resistance of the bacteria to both gastric acid and intestinal fluids [180].

Several factors affect the final number of viable bacterial cells, including the origin of the studied population and the type of diet [181]. In the United Kingdom population, the survival of the Shirota strain was assessed at 7.1 log CFU/mL when 65 mL of a probiotic beverage was consumed. In comparison, the Thai population consuming 80 mL of the probiotic beverage had a higher final number of live bacterial cells in the feces, which reached a level of 8.04 log CFU/mL. It can be assumed that the Thai diet, similar to the Mediterranean diet, is richer in dietary fiber and sources of vegetable fiber compared to the English diet, which ultimately affects the number of probiotic bacteria [182].

The *B. breve* strains, such as *B. breve* CCFM1025 and *B. breve* A1, are recognized as probiotics, but they also have psychobiotic effects. It has been proven that when administered at the right amount, these probiotic bacteria reduce the symptoms of AD and can also complement the preventive treatment of this disease. Such supplementation will be particularly beneficial in people who are genetically burdened with AD [183]. A study of the survival of *B. breve* bacteria was carried out by Adamberg et al. [184] from the Tallinn University of Technology. The authors observed that bacteria exposed to model digestive juices were largely resistant to the action of digestive enzymes and the low pH in the stomach. After 5 h of exposure to digestive juices, the number of bacteria decreased from 7.77 to 6.94 log CFU/mL and from 7.20 to 6.73 log CFU/mL. Another strain of *B. breve* also showed similar results. The number of bacterial cells decreased slightly after 5 h of exposure to digestive conditions from 7.53 to 6.94 log CFU/mL and from 7.20 to 6.99 log CFU/mL [184].

Unfortunately, there are a number of mental diseases, and most of these are associated with disturbed functions of the intestinal microbiota. According to recent data, IBS affects up to 10% of the population of North America and Europe [185]. Strains of the genus *Bifidobacterium* are particularly helpful in the probiotic treatment of this condition, as they alleviate unpleasant symptoms such as bloating, abdominal pain, diarrhea, or constipation. *Bifidobacterium* also has a positive effect on the microbiota–gut–brain axis and, by increasing the production of butyrate and serotonin, it modulates mood and may alleviate the accompanying stress and short-term inflammation. For example, *B. infantis* 35624 strain was shown to be highly effective in alleviating IBS symptoms. A randomized, double-blind study tested the survival of this strain during gastrointestinal transit. It was observed that a high number of bacteria survived gastrointestinal transit in patients with ulcerative colitis. Analysis of stool samples revealed the presence of *B. infantis* 35624 in the colon of study participants at 10^5^–10^8^ log CFU/mL. Such a high number of viable probiotic bacteria meets the therapeutic minimum, which indicates their potential health benefits to consumers [186].

A strain that is less known for its probiotic properties is *L. helveticus* NS8. This lactobacillus strain is widely used in the production of Italian and Swiss cheeses. The NS8 strain was isolated from the probiotic beverage kumis, which is very popular in Central Asia and Mongolia [187]. Subsequent reports indicated that this strain can inhibit the expression of interleukin-10, which is responsible for inducing an inflammatory cascade in the human body. The *L. helveticus* NS8 strain has been proven to be beneficial in the treatment of atopic dermatitis and has recently been recognized as a psychobiotic that can improve brain function as well as modulate cognitive functions. In vitro tests showed a high capacity of the strain to adhere to the surface of the intestinal epithelium of humans. Interestingly, this strain is characterized by a long period of intestinal colonization and the ability of self-aggregation, which facilitates its extended retention in the large intestine [188]. Together with commensal organisms present in the intestinal microbiota, *L. helveticus* NS8 can form biofilm-like structures, which ultimately allows the exclusion of other intestinal pathogens or inhibition of their development. The strain is also characterized by high survival during gastrointestinal transit; however, it exhibits low resistance to pancreatic enzymes [189].

## 6. Conclusions

Reports indicating a high prevalence of mental health and brain disorders urge researchers to develop methods to prevent or alleviate them. One of the key strategies is to influence the functioning of the microbiota–gut–brain axis, which connects the CNS and the digestive system through the vagus nerve, the dorsal root ganglia of the spinal cord, and the autonomic nervous system of the intestines. Data suggesting the indirect effect of the microbiota on the nervous system by modulating its action, the hypothalamic–pituitary–adrenal axis, neurotransmitters, and the synthesis of metabolites have led researchers to analyze the factors influencing this relationship. A promising approach to modulating the function of microbiota is to use psychobiotics, which are probiotic micro-organisms that affect the neuroimmune axis and cognitive processes, such as memory, learning, and general behavior.

One important factor affecting the effect of psychobiotics is their viability during processing, storage, and digestion, which must be at the level of 10^6^ to 10^9^ CFU/mL to achieve the desired therapeutic outcome. This paper has presented results showing the high viability of different strains of psychobiotics in fermented foods. However, the level of bacterial viability is closely related to the type of food matrix and the strain of the micro-organism; therefore, when designing food products rich in psychobiotics, it is necessary to analyze the survival of these bacteria and the factors influencing their survival. The most popular source of probiotic micro-organisms are dairy products, but there is a growing interest in plant-based fermented foods, which provide a promising matrix for the development of psychobiotics. Further research is needed to analyze the effectiveness of various types of plant matrices as carriers of psychobiotics.

A key factor to be analyzed while examining the functional properties of psychobiotics is their survival during the gastrointestinal passage. Studies conducted with animals and studies in laboratory conditions in model digestive systems have shown promising results regarding the therapeutic properties and viability of psychobiotics. Unfortunately, human research in this field is still limited. Therefore, it is necessary to broaden the existing knowledge about the survival of psychobiotics in the human digestive tract, their resistance to gastric and pancreatic enzymes, and their ability to colonize the microbiota. This will allow for an in-depth understanding of the nature and properties of psychobiotic micro-organisms and further analysis of their effect on improving brain and mental health.

## Figures and Tables

**Table 1:** Potential psychobiotic effect of probiotic bacterial strains.

Bacterial Strain	Study Model	Potential Psychobiotic Effects	References
*Lactiplantibacillus plantarum* C29	humans with mild cognitive impairment	improved combined cognitive functions	[18]
*L. plantarum* DR7	stressed adults	improved cognitive and memory functions and enhanced the serotonin pathway	[19]
*L. plantarum* P8	stressed adults	reduced scores of stress and anxiety; enhanced memory and cognitive traits	[20]
	stressed adults	enhanced the diversity of neurotransmitter-synthesizing/consuming species-level genome bins and the levels of some predicted microbial neuroactive metabolites reduced stress	[21]
*L. plantarum* PS128	mouse with 1-methyl-4-phenyl-1,2,3,6-tetrathydropyridine-induced Parkinson’s disease	inhibited neurodegenerative processes	[22]
	male children with autism spectrum disorder	ameliorated opposition/defiance behaviors and improved the total score of The Swanson, Nolan, and Pelham-IV	[23]
	adult IT specialists	improved self-perceived stress, overall job stress, job burden, cortisol level, general or psychological health, anxiety, depression, sleep disturbances, quality of life, and both positive and negative emotions	[24]
	patients with self-reported insomnia	decreases in Beck Depression Inventory-II scores, fatigue levels, brainwave activity, and awakenings during the deep sleep stage	[25]
*L. plantarum* ATCC 8014^TM^	Wistar rats	reduced the number of dead cells and increased acetylcholine in the brains of rats with Alzheimer disease (AD)	[26]
	male Wistar rats	ameliorated depression and anxiety-like behavior and cognitive performance improved serum and brain oxidative stress markers decreased oxidative stress in the hippocampus and amygdala	[27]
*L. plantarum* 299v	young adults under examination stress	prohibited increased levels of the stress marker cortisol during the examination period	[28]
	patients with major depressive disorder (MDD)	improvement in Attention and Perceptivity Test and in California Verbal Learning Test decrease in kynurenine concentration	[29]
*Limosilactobacillus reuteri* DSM 17938	mice	changed gut microbiota to modulate immune responses in murine experimental autoimmune encephalomyelitis	[30]
*Lactobacillus helveticus* CCFM1076	rats withvalproic acid-induced autism	restored neurotransmitter homeostasis by improving the balance of the 5-hydroxytryptamine system in the peripheral and central nervous systems, thereby ameliorating autistic-like behaviors	[31]
*L helveticus* NS8	chronic stress rats	improved chronic restraint stress-induced behavioral (anxiety and depression) and cognitive dysfunction	[32]
	rats with hyperammonemia	reduced the level of inflammatory markers, decreased serotonin metabolism, restored cognitive function, and improved anxiety-like behavior	[33]
*Lacticaseibacillus rhamnosus* JB-1	mice	reduced stress-induced corticosterone and anxiety- and depression-related behavior	[34]
	Wistar rats subjected to chronic unpredictable mild stress protocol	mitigated anxiety the level of brain metabolites was stable, with only the taurine level decreasing	[35]
*L. rhamnosus* GG	mice with induced obsessive-compulsive disorder (OCD)-like behavior	attenuating OCD-like behaviors	[36]
	middle-aged and older adults	improved cognitive performance because of improved total cognition score	[37]
	female mice	protective effect on gut microbiota relief of anxiety-like behavior in adult *L. rhamnosus* GG-colonized offspring	[38]
	drug-naive children and adolescents with a diagnosis of attention-deficit/hyperactivity disorder (ADHD)	improvement in the PedsQL Child Self-Report Total Score	[39]
*Lactobacillus gasseri CP2305*	male university Ekiden runners	prevented the stress-induced changes in the expression of genes related to mitochondrial functions	[40]
	young adults exposed to chronic stress	reduced anxiety and sleep disturbance	[41]
	Japanese medical students	improved sleep quality prevented increases in basal salivary cortisol release and expression of stress-responsive microRNAs normalized bowel habits under the stressful conditions	[42]
*Lacticaseibacillus casei* Shirota	male football players	decreased cognitive state anxiety scores, somatic state anxiety, and perceived stress scores	[43]
	healthy medical students under academic examination stress and rats with water avoidance stress	suppressed salivary cortisol levels and the incidence rate of physical symptoms in students suppressed water avoidance stress in rats	[44]
	patients with MDD or bipolar disorder (BD)	alleviated depression symptoms	[45]
*Bifidobacterium breve* A1	AD mice	prevented cognitive disorders	[46]
	elderly with mild cognitive impairment	improved cognitive function	[47]
	patients with schizophrenia	improved anxiety and depressive symptoms	[48]
*B. breve* CCFM1025	C57BL/6J mice	reduced depression- and anxiety-like behaviors	[49]
	C57BL/6J mice	decreased depressive-like behaviors and neurological abnormalities of chronically stressed mice reshaped the gut microbiome of chronically stressed mice	[50]
	Mice with AD	improved synaptic plasticity and increased the concentrations of brain-derived neurotrophic factor, fibronectin type III domain-containing protein 5, and postsynaptic density protein 95	[51]
	pregnant mice	protected the offspring from maternal separation-induced neurobiological and gastrointestinal disorders such as depression-like behavior and delayed defecation	[52]
*B. longum* NCC3001	adults with irritable bowel syndrome (IBS)	reduced depression, but not anxiety scores, and increased quality of life	[53]
*B. longum* 1714^TM^	healthy volunteers	reduced stress and improved memory	[54]
	healthy volunteers	increased social stress	[55]
*B. infantis* 35624	adult rats	normalization of the immune response, reversal of behavioral deficits, and restoration of basal noradrenaline concentrations in the brainstem	[56]
*B. bifidum* ATCCVR 29521	Wistar rats	reduced the number of dead cells and increased acetylcholine in the brains of rats with AD	[26]
*Bacillus coagulans* MTCC 5856	patients diagnosed for major depressive disorder with IBS	the improvement in depression and IBS symptoms	[57]
*Clostridium butyricum* MIYAIRI 588	adult patients diagnosed with treatment-resistant major depressive disorder	significant improvement in depression (in combination with antidepressants)	[58]

**Table 2:** The survival of psychobiotics in fermented foods.

Food Category	Type of Fermented Food Product	Micro-Organisms Used in Fermentation	Viability of Micro-Organisms after Fermentation [log CFU/mL]	Viability of Micro-Organisms after Storage		References
				Storage Time [Days]	Population Viability [log CFU/mL]	
Dairy products	Yogurt	*L. plantarum* 299v	8.0	56	7.5	[140]
	Cheese		9.5	56	9.0	[140]
	Ice cream	*L. plantarum* ATCC 8014	7.5	60	7.6	[141]
	Cow milk	*L. rhamnosus* GG	9.0	-	-	[142]
	Cow milk		8.0	-	-	[143]
	Goat milk		9.5	-	-	[144]
	Ice cream		8.3	90	7.3	[145]
	Sheep milk yogurt		7.5–8.0	21	7.4–7.8	[146]
	Milk-based dessert with cranberry sauce	*L. casei* Shirota	8.0	21	7.3	[147]
	Cow Milk		8.0	31	8.0	[148]
	Cow Milk		8.8	28	7.8	[149]
	Pudding		7.3	20	9.0	[150]
	Skimmed milk with milk protein concentrate	*B. coagulans* MTCC 5856	8.4	60	8.1	[151]
Plant products	Watermelon juice	*L. plantarum* ATCC 8014	8.8	14	11.0	[152]
	Apple juice		11.0–11.5	42	7.7–8.6	[153]
	Sesame	*L. plantarum* P8	8.6	-	-	[154]
	Whole soybeans		10.5	-	-	[155]
	Bread		5.0	5	8.0	[156]
	Sourdough	L. plantarum 299v	7.9	-	-	[157]
	Dark chocolate		8.2	360	5.5	[158]
	Tomato juice	*L. reuteri* DSM 17938	7.1	28	5.7	[159]
	Coconut milk		8.6	30	8.6	[160]
	Blueberry pomace	*L. rhamnosus* GG	11.6	-	-	[161]
	Hazelnut milk		7.9	28	8.3	[162]
	Pineapple and jussarajuice		7.2	28	7.7	[163]
	Teff-based beverage		8.1	25	7.8	[164]
	Coffee brews		7.8	49	7.0	[165]
	Coconut water	*B. coagulans* MTCC 5856	9.73	-	-	[166]

## Data Availability

Data sharing not applicable.
